# The efficacy and safety of different pharmacological interventions for patients with advanced biliary tract cancer: A network meta-analysis

**DOI:** 10.18632/oncotarget.20445

**Published:** 2017-08-24

**Authors:** Xin-Fang Sun, Zhi-Kuan He, Jin-Ping Sun, Quan-Xing Ge, Er-Dong Shen

**Affiliations:** ^1^ Department of Gastroenterology, Henan University Huaihe Hospital, Kaifeng, 475000, Henan, China; ^2^ Department of Oncology, The First People’s Hospital of Yueyang, Yueyang, 414000, Hunan, China

**Keywords:** biliary tract cancer, chemotherapy treatments, network meta-analysis, efficacy, safety

## Abstract

Biliary tract cancer (BTC) is the second common cancer in liver cancer. Chemotherapy is the mainstay of treatments for patients with advanced or metastatic disease, while fluorouracil (FU)-based and gemcitabine (GEM)-based treatments are most widely applied. This NMA aimed to figure out whether the addition of platinum (PLA) and target agents (TAR) can influence the efficacy and safety of standard chemotherapy. Network meta-analysis (NMA) was conducted based on the records from PubMed, Embase and Cochrane. Eligible data was extracted from available qualified trials and outcomes. Software R 3.2.3 and STATA 13.0 were used to conduct the Bayesian NMA, calculating odds ratios (ORs) and hazard ratios (HRs) with 95% credible interval (CrI) to evaluate different treatments.Almost all treatments were superior to best supportive care (BSC) and FU in terms of 1-OS, 2-OS and 1-PFS. GEM+PLA and GEM+PLA+TAR exhibited better efficacy than most treatments in 1-OS, 2-OS and 1-PFS, and yielded better results than BSC and GEM+FU in terms of 2-PFS. Most drug-containing treatments reported higher overall response rate (ORR) than BSC. GEM and GEM+FU were associated with a higher risk of neutropenia and thrombocytopenia compared to FU, FU+PLA and GEM+PLA. No statistical difference was detected in terms of nausea and vomiting.GEM+PLA and GEM+PLA+TAR were both efficacious and were associated with fewer adverse events. In conclusion, the addition of PLA can significantly improve the efficacy of FU and GEM-based treatments, and the addition of TAR to GEM+PLA can contribute to further improvement, but with a mild increase of adverse events.

## INTRODUCTION

Biliary tract cancer (BTC), including gallbladder cancer (GBC), intrahepatic cholangiocarcinoma (ICC), extrahepatic cholangiocarcinoma (ECC) and hilar cholangiocarcinoma (HCC), is the second common tumor in liver cancer reports. Although not popular in western countries, the occurrence rate of BTC was particularly high in Southeast Asia [1]. The only way to cure BTC is surgical resection. However, as it is hard to identify at its early stage, most patients are diagnosed with unresectable advanced BTC, with a median overall survival of only 16 months [2]. According to Yang et al. [3], 70% ICC cases are unresectable at the time of diagnosis.

Chemotherapy is now the mainstay of treatment for BTC, and gemcitabine (GEM) and fluorouracil (FU) based chemotherapy treatments have been proved to be particularly effective compared to best supportive care (BSC) [4]. A study conducted by Ducreux *et al.* showed that the response rate of patients treated with 5-FU based chemotherapy reached 30% [5]. Moreover, it was found recently that the addition of platinum (PLA) to standard chemotherapy can further improve survival without substantial toxicity. For example, a study conducted by Valle *et al.* reported that the addition of cisplatin (CIS) to GEM can greatly improve overall survival (OS) and progression-free survival (PFS) of patients with advanced BTC [6].

In addition to platinum drugs, targeted drugs (TAR) were also introduced in standard chemotherapy to improve efficacy while reducing side effects. Targeted agents, such as epidermal growth factor receptor (EGFR) and vascular endothelial growth factor (VEGF) regulated the growth and proliferation of biliary cell, thus exhibited encouraging antitumor activity [7]. A study conducted by Gruenberger *et al.* suggested that the addition of cetuximab to GEM plus oxaliplatin (OXA) yielded better results in terms of response rate and adverse events [8].

However, despite the reported advantages of additional drugs, some studies did not seem to support those results. For instance, a trial conducted by Chen *et al.* reported the absence of statistical difference between targeted drugs plus GEM+PLA and GEM+PLA [9]. Even the effect of platinum was to some extent denied according to the results of another study [10]. Thus, no definite conclusion was drawn. Moreover, there are no references to facilitate choices among different chemotherapy treatments. In order to address the issues above, this NMA was designed to evaluate different chemotherapy treatments from mainly randomized controlled trials (RCTs) based on their performance on efficacy and safety outcomes targeting patients with advanced BTC.

## RESULTS

### Literature selection results

**Figure 1:**
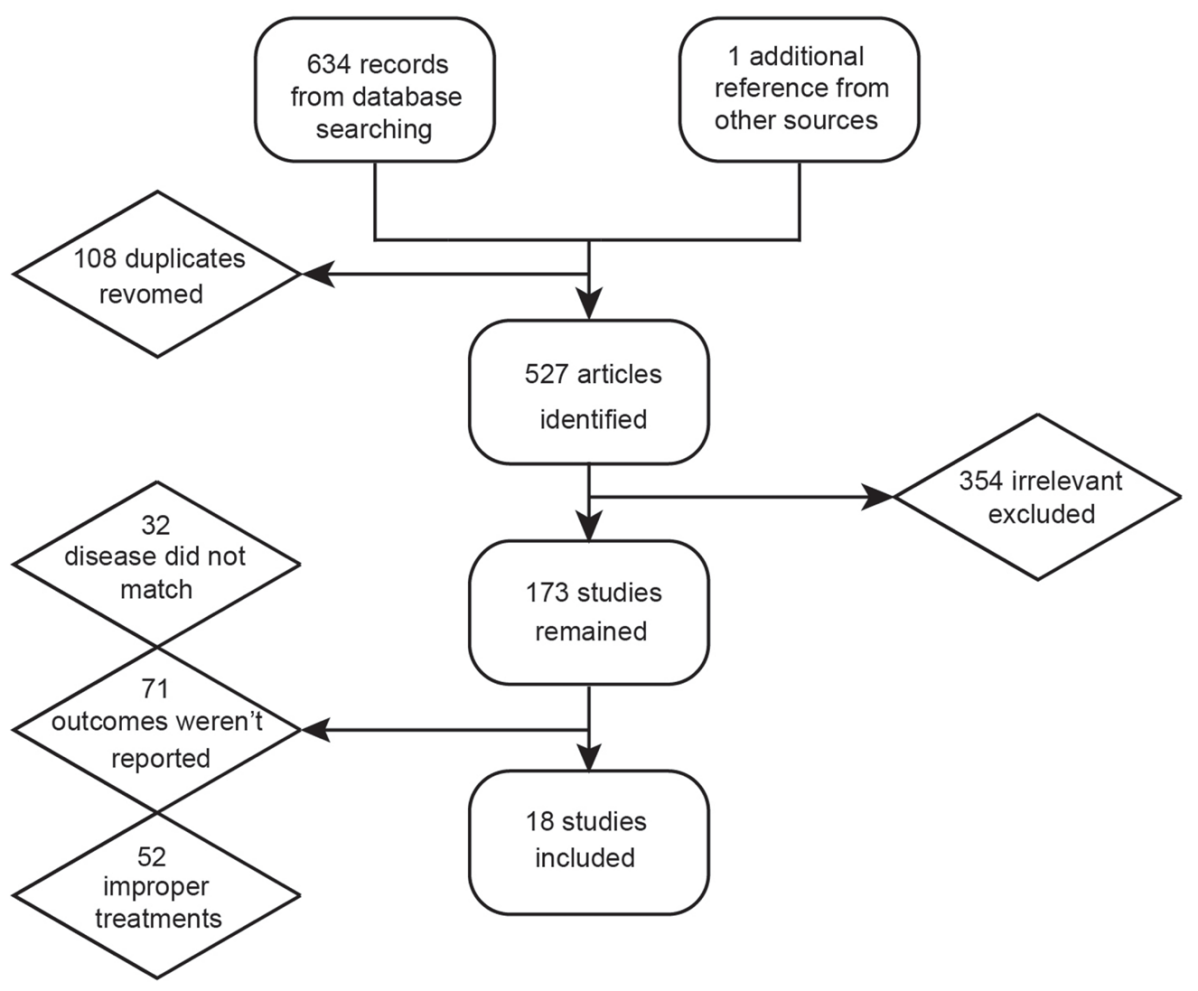
Flow chart of study selection

**Figure 2:**
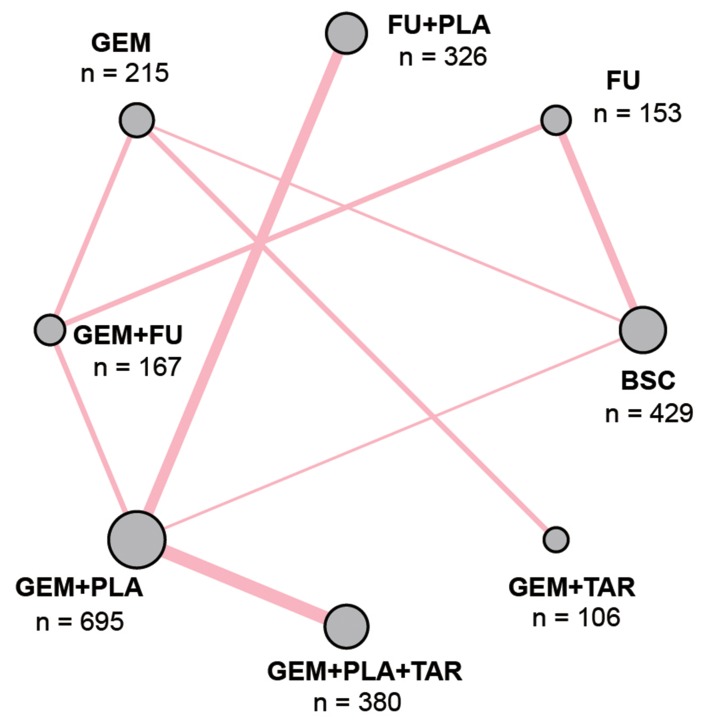
Network plot of included studies The size of each circle represents the sum of the samples; a solid line represents the direct comparison of the two therapies and the width of each line stands for the number of two-armtrials comparison. Abbreviation: GEM, gemcitabine; PLA, Platinum; FU, fluorouracil; TAR, target agents; BSC, best supportive care.

Initially, 634 records were retrieved through the electronic databases mentioned above and one additional record was found from reviews. As shown in Figure 1, after removing 108 duplicates and 354 irrelevant records, investigators read the full text of the remaining articles and excluded another 155 articles due to: 1) the disease did not match; 2) relevant outcomes were not reported and 3) treatments cannot form a network (insufficient network). In the end, 18 records with a total of 2,471 patients were included in this NMA [9, 11–27]. The network plot illustrated the comparison among 7 drug-including treatments and BSC and all the drug-including treatments were based on either FU or GEM (Figure 2). As shown in Table 1 , most trials included a follow-up period of more than 2 years, which to some degree guarantees the reliability of results in this NMA.

**Table 1:** Characteristics of included studies of biliary tract cancer treatment

Study	Country	Design	Follow-up	Treatment 1				Treatment 2				Outcomes
				Size	Treatment	Age (range)	Man (%)	Size	Treatment	Age (range)	Man (%)	
Chen, 2015	China	RCT	27	60	GEM+PLA	59(32-80)	50	62	GEM+PLA+TAR	61(32-78)	45	(1)(2)(3)(4)(5)(6)(7)(8)
Fiteni, 2014	France	Retro	24	44	GEM+PLA	-	66	20	GEM+FU	-	45	(1)(2)(3)(4)(5)(6)(7)(8)
Kang, 2012	Korea	RCT	36	49	GEM+PLA	59(32-77)	63	47	FU+PLA	60(36-77)	92	(1)(2)(3)(4)(5)(6)(7)(8)
Lee, 2015	Korea	Retro	24	49	GEM+PLA	65(45-81)	63.3	44	FU+PLA	65(39-80)	68.2	(1)(2)(3)(4)(5)(6)(7)(8)
Lee, 2012	Korea	RCT	18	133	GEM+PLA	61(55-68)	59	135	GEM+PLA+TAR	59(54-66)	67	(1)(2)(3)(4)(5)(6)(7)(8)
Leone, 2016	Italy	RCT	42	44	GEM+PLA	64(37-79)	34	45	GEM+PLA+TAR	64(47-79)	37.7	(1)(2)(3)(4)(5)(6)(7)(8)
Li, 2016	China	RCT	24	25	GEM	-	-	25	GEM+FU	-	-	(1)(2)(3)(4)(5)(6)(7)(8)
				25	FU	-	-	25	GEM+FU	-	-	(1)(2)(3)(4)(5)(6)(7)(8)
Malka, 2014	France	RCT	34	74	GEM+PLA	62(39-75)	57	76	GEM+PLA+TAR	61(35-75)	57	(1)(2)(3)(4)(5)(6)(7)(8)
Moehler, 2014	Germany	RCT	31	49	GEM+TAR	64(44-83)	59	48	GEM	65(36-84)	52	(1)(2)(3)(4)(5)(6)(7)(8)
Morizane, 2013	Japan	RCT	24	51	GEM+FU	66(39-78)	52.9	50	FU	63(49-79)	56	(1)(2)(3)(5)(6)(7)(8)
Phelip, 2014	France	RCT	30	18	FU+PLA	70(53-80)	39	16	GEM+PLA	75(54-81)	50	(1)(2)(7)
Rogers, 2014	USA	Retro	60	11	GEM+PLA	-	-	16	GEM+FU	-	-	(1)(2)
Santoro, 2015	Italy	RCT	33	57	GEM+TAR	(55-74)	53.4	52	GEM	(55-73)	44.6	(1)(2)(3)(4)(5)(6)(7)
Sasaki, 2013	Japan	RCT	24	30	GEM+FU	68(47-83)	53	32	GEM	75(55-86)	63	(1)(2)(3)(4)(5)(6)(7)(8)
Sharma, 2010	India	RCT	27	28	FU	47	18	27	BSC	51	22	(1)(2)(3)(4)(5)
				26	GEM+PLA	49	19	27	BSC	51	22	(1)(2)(3)(4)(5)
Valle, 2015	UK	RCT	36	62	GEM+PLA+	68(60-73)	55	62	GEM+PLA	65(60-73)	45	(1)(2)(3)(4)(5)(6)(7)(8)
Woo, 2014	Korea	Retro	45	127	GEM+PLA	62(35-76)	56.7	217	FU+PLA	58(27-82)	62.8	(1)(2)(3)(4)(5)(6)
Yonemoto, 2007	Japan	Retro	52	30	FU	-	43	125	BSC	-	61	(1)(2)(3)(4)
				20	FU	-	65	125	BSC	-	61	(1)(2)(3)(4)
				58	GEM	-	52	125	BSC	-	61	(1)(2)(3)(4)

Abbreviation: RCT, randomized controlled trial; Retro, retrospective trial; GEM, gemcitabine; PLA, Platinum; FU, fluorouracil; TAR, target agents; BSC, best supportive care; Outcomes: (1) overall survival; (2) progression-free survival; (3) overall response rate; (4) disease control rate; (5) vomiting; (6) nausea; (7) neutropenia; (8) thrombocytopenia

### Overall survival

**Figure 3:**
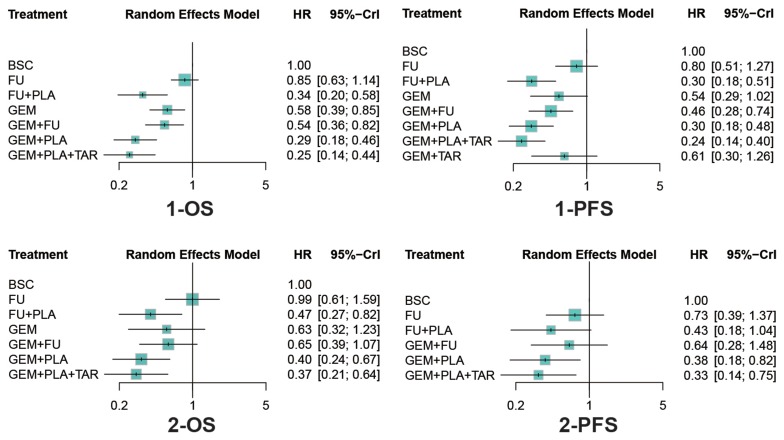
Forest plots of survival outcomes Hazard ratios (HRs) and 95% credible interval (CrIs) indicate the relative efficacy under the corresponding endpoint. Abbreviation: GEM, gemcitabine; PLA, Platinum; FU, fluorouracil; TAR, target agents; BSC, best supportive care; OS, overall survival; PFS, progression free survival.

Results concerning OS were presented in Table 2 and Figure 3. In terms of 1-OS, except for FU, all treatments exhibited better efficacy than BSC (FU+PLA: HR=0.34, 95%CrI=0.20-0.58; GEM: HR=0.58, 95%CrI=0.39-0.85; GEM+FU: HR=0.54, 95%CrI= 0.36-0.82; GEM+PLA: HR=0.29, 95%CrI=0.18-0.46; GEM+PLA+TAR: HR=0.25, 95%CrI=0.14-0.44). Besides, GEM+PLA and GEM+PLA+TAR were superior to most treatments, including GEM+FU (HR=0.53, 95%CrI=0.39-0.72; HR=0.46, 95%CrI=0.30-0.72, respectively), GEM (HR=0.50, 95%CrI=0.30-0.83; HR=0.44, 95%CrI=0.24-0.79), FU (HR=0.34, 95%CrI=0.21-0.54; HR=0.30, 95%CrI=0.17-0.53), while FU turned out to be the worst choice for its inferiority to most treatments. As for 2-OS, treatments containing PLA showed better performance than BSC (FU+PLA: HR=0.47, 95%CrI=0.27-0.82; GEM+PLA: HR=0.40, 95%CrI=0.24-0.67; GEM+PLA+TAR: HR=0.37, 95%CrI=0.21-0.64). GEM+PLA and GEM+PLA+TAR yielded better results than GEM (HR=0.62, 95%CrI=0.49-0.79; HR=0.57, 95%CrI=0.40-0.80, respectively) and FU (HR=0.41, 95%CrI=0.26-0.62; HR=0.37, 95%CrI=0.23-0.60). Similar to 1-OS, FU exhibited the worst performance of all drug-containing treatments.

**Table 2:** Network comparison of prognostic and response outcomes of different therapies for biliary tract cancer treatments

**1-OS**	**BSC**	0.99(0.61,1.59)	0.47(0.27,0.82)	0.63(0.32,1.23)	0.65(0.39,1.07)	0.40(0.24,0.67)	0.37(0.21,0.64)		**2-OS**
	0.85(0.63,1.14)	**FU**	0.48(0.29,0.77)	0.63(0.35,1.14)	0.65(0.45,0.95)	0.41(0.26,0.62)	0.37(0.23,0.60)		
	0.34(0.20,0.58)	0.40(0.23,0.69)	**FU+PLA**	1.33(0.76,2.32)	1.37(0.98,1.92)	0.85(0.68,1.07)	0.78(0.56,1.08)		
	0.58(0.39,0.85)	0.68(0.43,1.07)	1.72(0.96,3.07)	**GEM**	0.97(0.62,1.51)	0.62(0.49,0.79)	0.57(0.40,0.80)		
	0.54(0.36,0.82)	0.64(0.42,0.97)	1.62(1.07,2.44)	0.94(0.60,1.47)	**GEM+FU**	1.56(0.94,2.60)	0.91(0.72,1.16)		
	0.29(0.18,0.46)	0.34(0.21,0.54)	0.85(0.65,1.12)	0.50(0.30,0.83)	0.53(0.39,0.72)	**GEM+PLA**	1.71(0.98,3.00)		
	0.25(0.14,0.44)	0.30(0.17,0.53)	0.75(0.50,1.13)	0.44(0.24,0.79)	0.46(0.30,0.72)	0.88(0.65,1.20)	**GEM+PLA+TAR**		
**1-PFS**	**BSC**	0.73(0.39,1.37)	0.43(0.18,1.04)	-	0.64(0.28,1.48)	0.38(0.18,0.82)	0.33(0.14,0.75)	-	**2-PFS**
	0.80(0.51,1.27)	**FU**	0.59(0.20,1.74)	-	0.88(0.31,2.51)	0.52(0.19,1.41)	0.45(0.16,1.27)	-	
	0.30(0.18,0.51)	0.37(0.26,0.53)	**FU+PLA**	-	1.49(0.88,2.54)	0.88(0.58,1.34)	0.76(0.45,1.27)	-	
	0.54(0.29,1.02)	0.68(0.42,1.09)	1.82(1.14,2.92)	**GEM**	-	-	-	-	
	0.46(0.28,0.74)	0.57(0.44,0.73)	1.53(1.19,1.96)	0.84(0.56,1.25)	**GEM+FU**	0.59(0.43,0.82)	0.51(0.33,0.79)	-	
	0.30(0.18,0.48)	0.37(0.28,0.49)	0.99(0.80,1.22)	0.54(0.36,0.83)	0.65(0.56,0.75)	**GEM+PLA**	0.86(0.64,1.16)	-	
	0.24(0.14,0.40)	0.30(0.21,0.42)	0.80(0.61,1.05)	0.44(0.28,0.69)	0.53(0.42,0.66)	0.81(0.68,0.96)	**GEM+PLA+TAR**		
	0.61(0.30,1.26)	0.77(0.42,1.38)	2.05(1.14,3.70)	1.13(0.79,1.61)	1.35(0.79,2.29)	2.07(1.19,3.60)	2.56(1.44,4.57)	**GEM+TAR**	
**ORR**	**BSC**	0.32(0.08,1.17)	1.09(0.28,4.53)	2.61(0.57,12.18)	1.13(0.34,3.82)	1.90(0.53,7.32)	0.98(0.12,7.54)	1.45(0.24,8.94)	**DCR**
	4.39(0.57,56.26)	**FU**	3.42(0.68,18.73)	8.00(2.08,35.52)	3.56(0.76,16.12)	5.93(1.23,29.96)	3.00(0.43,22.20)	4.44(0.84,25.79)	
	27.66(3.25,572.49)	6.36(0.59,94.63)	**FU+PLA**	2.39(0.53,10.49)	1.03(0.53,1.82)	1.73(0.78,3.71)	0.90(0.11,6.36)	1.34(0.22,7.69)	
	14.88(1.73,214.86)	3.39(1.14,11.02)	0.54(0.04,5.10)	**GEM+FU**	0.43(0.11,1.67)	0.73(0.17,3.06)	0.37(0.09,1.45)	0.55(0.21,1.45)	
	20.29(2.64,361.41)	4.66(0.49,57.97)	0.74(0.29,1.58)	1.35(0.16,15.49)	**GEM+PLA**	1.68(1.07,2.80)	0.87(0.12,5.81)	1.30(0.25,6.96)	
	40.85(4.85,780.55)	9.39(0.91,126.47)	1.49(0.48,3.94)	2.72(0.30,34.12)	1.99(1.08,3.78)	**GEM+PLA+TAR**	0.51(0.07,3.56)	0.77(0.14,4.31)	
	10.70(0.64,252.14)	2.39(0.31,19.89)	0.37(0.02,6.75)	0.71(0.12,4.01)	0.51(0.03,8.41)	0.25(0.01,4.53)	**GEM+TAR**	1.51(0.56,4.06)	
	7.24(0.59,129.02)	1.62(0.30,8.85)	0.26(0.01,3.32)	0.48(0.13,1.62)	0.35(0.02,4.06)	0.18(0.01,2.18)	0.67(0.20,2.23)	**GEM**	

Abbreviation: BSC, best supportive care; GEM, gemcitabine; OXA, Oxaliplatin; CIS, Cisplatin; PLA, Platinum; FU, fluorouracil; TAR, targeted drugs; OS, overall survival; PFS, progression free survival; ORR, overall response rate; DCR, disease control rate. The data is in the form of hazard ratio (HR) and corresponding 95% credible intervals (CrI) for 1-OS, 2-OS, 1-PFS and 2-PFS and in the form of odds ratio (OR) and corresponding 95% CrI for ORR and DCR.

### Progression free survival

Also as was shown in Table 2 and Figure 3, four drug-containing treatments, including FU+PLA, GEM+FU, GEM+PLA and GEM+PLA+TAR showed better performance than BSC (HR=0.30, 95%CrI=0.18-0.51; HR=0.46, 95%CrI=0.28-0.74; HR=0.30, 95%CrI=0.18-0.48; HR=0.24, 95%CrI=0.14-0.40) with respect to 1-PFS. Plus, treatments containing PLA, including FU+PLA, GEM+PLA and GEM+PLA+TAR were superior to those without PLA, including FU (HR=0.37, 95%CrI=0.26-0.53; HR=0.37, 95%CrI=0.28-0.49; HR=0.30, 95%CrI=0.21-0.42), GEM (HR=0.55, 95%CrI=0.34-0.88; HR=0.54, 95%CrI=0.36-0.83; HR=0.44, 95%CrI=0.28-0.69), GEM+FU (HR=0.66, 95%CrI=0.51-0.84; HR=0.65, 95%CrI=0.56-0.75; HR=0.53, 95%CrI=0.42-0.66) and GEM+TAR (HR=0.49, 95%CrI=0.27-0.88; HR=0.48, 95%CrI=0.28-0.84; HR=0.39, 95%CrI=0.22-0.70). As for 2-PFS, GEM+PLA and GEM+PLA+TAR showed higher efficacy than BSC (HR=0.38, 95%CrI=0.18-0.82; HR=0.33, 95%CrI=0.14-0.75) and GEM+FU (HR=0.59, 95%CrI=0.43-0.82; HR=0.51, 95%CrI=0.33-0.79).

### Overall response rate and disease control rate

**Figure 4:**
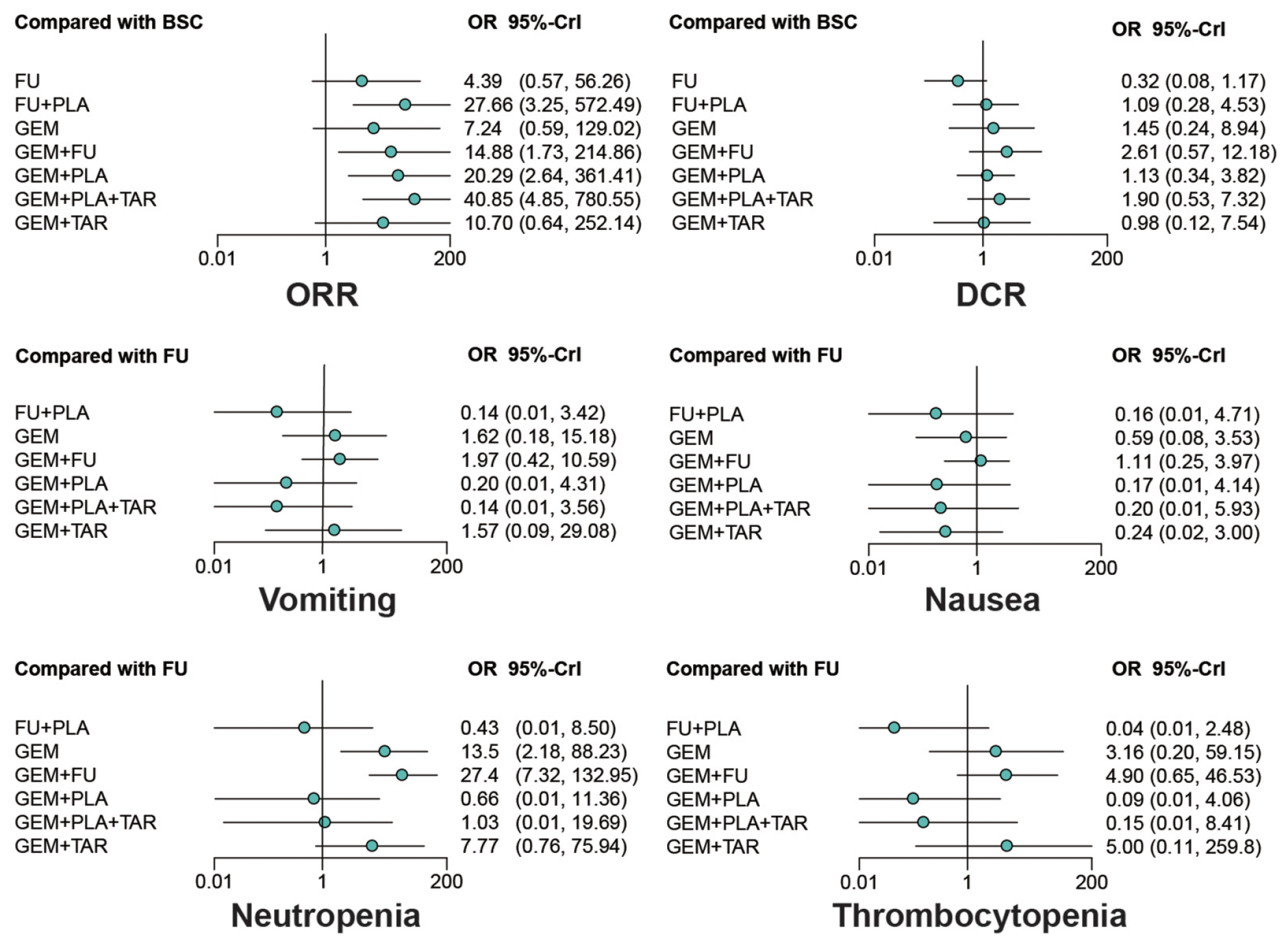
Forest plots of overall response rate (ORR), disease control rate (DCR) and adverse events Odds ratios (ORs) and 95% credible interval (CrIs) indicate the relative efficacy or safety under the corresponding endpoint. Abbreviation: GEM, gemcitabine; PLA, Platinum; FU, fluorouracil; TAR, target agents; BSC, best supportive care; ORR, overall response rate; DCR, disease control rate.

In terms of overall response rate (ORR) (Table 2 and Figure 4), most treatments, including FU+PLA, GEM+FU, GEM+PLA and GEM+PLA+TAR were significantly better than BSC (OR=27.66, 95%CrI=3.25-572.49; OR=14.88, 95%CrI=1.73-214.86; OR=20.29, 95%CrI=2.64-361.41; OR=40.85, 95%CrI=4.85-780.55). Besides, the addition of TAR to GEM+PLA improved the corresponding ORR (OR=1.99, 95%CrI=1.08-3.78). With respect to disease control rate (DCR), GEM+FU and GEM+PLA+TAR exhibited better results than FU (HR=8.00, 95%CrI=2.08-35.52; OR=5.93, 95%CrI=1.23-29.96), and similarly, TAR improved the efficacy of GEM+PLA (OR=1.68, 95%CrI=1.07-2.80).

### Adverse events (grade≥3)

As shown in Table 3 and Figure 4, GEM and GEM+FU were more likely to cause neutropenia compared with FU (OR=13.46, 95%CrI=2.18-88.23; OR=27.39, 95%CrI=7.32-132.95) and FU+PLA (OR=31.50, 95%CrI=1.97-1863.11; OR=65.37, 95%CrI=5.050-3261.69), while the addition of PLA seemed to offset this effect (GEM+PLA vs. GEM: OR=0.05, 95%CrI=0.01-0.67; GEM+PLA vs. GEM+FU: OR=0.02, 95%CrI=0.01-0.25; GEM+PLA+TAR vs. GEM+FU: OR=0.04, 95%CrI=0.01-0.44). As for thrombocytopenia, FU+PLA significantly reduced the toxicity compared to GEM (OR=0.01, 95%CrI=0.01-0.64), GEM+FU (OR=0.01, 95%CrI=0.01-0.25) and GEM+TAR (OR=0.01, 95%CrI=0.01-0.86), while GEM+FU increased the risk compared to GEM+PLA (OR=53.52, 95%CrI=2.66-2465.13) and GEM+PLA+TAR (OR=33.78, 95%CrI=1.23-1826.21). Besides, no significant results were detected with respect to nausea and vomiting.

**Table 3:** Network comparison of adverse events of different therapies for biliary tract cancer

**Neutropenia**	**FU**	0.16(0.00,4.71)	0.59(0.08,3.53)	1.11(0.25,3.97)	0.17(0.00,4.14)	0.20(0.00,5.93)	0.24(0.02,3.00)	**Nausea**
	0.43(0.01,8.50)	**FU+PLA**	3.71(0.12,149.90)	6.69(0.30,239.85)	1.04(0.38,2.75)	1.26(0.30,5.05)	1.52(0.03,90.02)	
	13.46(2.18,88.23)	31.50(1.97,1863.11)	**GEM**	1.82(0.50,7.54)	0.28(0.01,7.39)	0.34(0.01,10.28)	0.42(0.07,2.14)	
	27.39(7.32,132.95)	65.37(5.05,3261.69)	2.05(0.66,6.82)	**GEM+FU**	0.16(0.00,2.94)	0.19(0.01,4.10)	0.23(0.02,1.86)	
	0.66(0.01,11.36)	1.49(0.63,4.18)	0.05(0.00,0.67)	0.02(0.00,0.25)	**GEM+PLA**	1.21(0.42,3.32)	1.46(0.04,79.04)	
	1.03(0.01,19.69)	2.36(0.78,8.67)	0.08(0.00,1.21)	0.04(0.00,0.44)	1.58(0.77,3.39)	**GEM+PLA+TAR**	1.22(0.03,75.94)	
	7.77(0.76,75.94)	18.17(0.76,1366.49)	0.57(0.14,2.18)	0.27(0.04,1.60)	11.82(0.58,788.40)	7.54(0.33,544.57)	**GEM+TAR**	
**Thrombocytopenia**	**FU**	0.14(0.00,3.42)	1.62(0.18,15.18)	1.97(0.42,10.59)	0.20(0.00,4.31)	0.14(0.00,3.56)	1.57(0.09,29.08)	**Vomiting**
	0.04(0.00,2.48)	**FU+PLA**	12.55(0.55,713.37)	14.59(0.96,692.29)	1.49(0.58,4.22)	1.02(0.26,4.06)	11.70(0.32,1130.03)	
	3.16(0.20,59.15)	76.71(1.55,7863.60)	**GEM**	1.21(0.25,5.47)	0.12(0.00,2.66)	0.08(0.00,2.16)	0.96(0.14,6.55)	
	4.90(0.65,46.53)	120.30(3.97,8518.54)	1.58(0.25,10.38)	**GEM+FU**	0.10(0.00,1.32)	0.07(0.00,1.06)	0.79(0.07,9.68)	
	0.09(0.00,4.06)	2.16(0.41,14.88)	0.03(0.00,1.02)	0.02(0.00,0.38)	**GEM+PLA**	0.68(0.26,1.73)	7.77(0.22,639.06)	
	0.15(0.00,8.41)	3.42(0.47,37.34)	0.05(0.00,2.10)	0.03(0.00,0.81)	1.58(0.49,5.70)	**GEM+PLA+TAR**	11.82(0.28,1118.79)	
	5.00(0.11,259.82)	123.97(1.16,26108.08)	1.60(0.11,22.65)	1.00(0.04,25.79)	55.70(0.67,8103.08)	35.16(0.34,5710.15)	**GEM+TAR**	

Abbreviation: GEM, gemcitabine; PLA, Platinum; FU, fluorouracil; TAR, targeted drugs. The data is in the form of odds ratio (OR) and corresponding 95% credible intervals (CrI).

### Ranking

According to SUCRAs presented in Table 4 , GEM+PLA+TAR ranked the first in all survival terms, while FU+PLA showed an excellent control of adverse events. GEM+PLA also exhibited relative better performance in all terms. Besides, GEM+FU was outstanding in terms of DCR, GEM+TAR showed a good control of nausea while FU was associated with relative low risk of neutropenia. Generally, treatments containing PLA showed superiority concerning both efficacy and safety outcomes and were more recommended than those without PLA.

**Table 4:** SUCRA of different treatments for all outcomes in biliary tract cancer

	1-OS	1-PFS	2-OS	2-PFS	ORR	DCR	Vomiting	Nausea	Neutropenia	Thrombocytopenia
**BSC**	0.023	0.042	0.104	0.071	0.029	0.416	-	-	-	-
**FU**	0.155	0.152	0.099	0.316	0.226	0.049	0.411	0.234	0.692	0.483
**FU+PLA**	0.694	0.787	0.660	0.631	0.748	0.437	0.841	0.712	0.907	0.945
**GEM**	0.398	0.409	0.439	-	0.351	0.595	0.271	0.401	0.193	0.276
**GEM+FU**	0.433	0.524	0.404	0.303	0.630	0.881	0.182	0.175	0.029	0.145
**GEM+PLA**	0.848	0.790	0.851	0.757	0.603	0.448	0.661	0.706	0.768	0.797
**GEM+PLA+TAR**	0.949	0.991	0.945	0.923	0.899	0.778	0.834	0.614	0.582	0.666
**GEM+TAR**	-	0.304	-	-	0.515	0.397	0.300	0.657	0.330	0.188

Abbreviation: BSC, best supportive care; GEM, gemcitabine; OXA, Oxaliplatin; CIS, Cisplatin; PLA, Platinum; FU, fluorouracil; TAR, targeted drugs; OS, overall survival; PFS, progression free survival; ORR, overall response rate; DOR, disease control rate.

## DISCUSSION

This NMA was designed to address the problem whether the addition of platinum and targeted drugs to GEM or FU based chemotherapy regimens can improve efficacy while reduce toxicity, and furthermore, serve as a reference to clinical practice. A total of 7 drug-containing treatments and BSC were compared with respect to their efficacy and safety.

First of all, according to SUCRA rankings, the addition of PLA to standard chemotherapy did improve the effect on survival and moreover, lower the risk of adverse events, which is consistent to the results of most studies. For example, a meta-analysis conducted by Yang *et al.* [28] reported that all survival outcomes were significantly more favorable for patients treated with GEM+PLA than those with GEM alone, while another systematic review conducted by Park [29] suggested that the adverse events associated with GEM+PLA were generally more acceptable and manageable. However, according to this NMA, some exceptions existed. For instance, the addition of PLA to GEM seemed to dilute DCR, while the addition of PLA to GEM+TAR appeared to increase the control of disease, which might be caused by the lack of direct evidence.

Secondly, the effect of TAR was also confirmed in this NMA. According to SUCRA, the addition of TAR to GEM+PLA improved all survival outcomes and was associated with increased ORR, DCR and higher risk of adverse effects. Similar results were reported in previous studies. For example, an RCT conducted by Chen *et al.* [9] concluded that the addition of cetuximab to GEM+PLA could significantly improve PFS. However, the addition of TAR to GEM+PLA seemed to mildly increase the adverse events of chemotherapy, and this effect was also confirmed in previous studies. A trial designed by Valle [13] reported that patients treated with cediranib plus GEM+PLA had more adverse events. Thus, the toxicity of TAR should be noted before putting it into use. Moreover, due to the lack of evidence, the effect of TAR added to GEM remained unclear, and further investigations should be made to reach a definite conclusion.

In addition to evaluating the role of TAR and PLA, some other observations were made in this NMA. Firstly, treatments containing GEM were more efficacious than those with FU. For instance, GEM and GEM+PLA yielded more desirable survival outcomes than FU and FU+PLA respectively. In the meantime, treatments containing FU were associated with less adverse events. Similar results were obtained in other articles. A study conducted by Kang [25] recorded that more adverse events were reported from patients treated with GEM+PLA than those with FU+PLA. Another study designed by Lee [15] suggested that GEM+PLA resulted in a superior response rate compared to FU+PLA. Secondly, the addition of FU to GEM showed little superiority to GEM in both survival outcomes and adverse events, which was also confirmed in a previous study [12].

As the first NMA comprehensively judged chemotherapy treatments of BTC, we systematically analyzed the efficacy and adverse effect of several treatments. Although conducted as meticulously as possible, this NMA still had some limitations. First of all, the treatment FU actually referred to different FU-based or FU-related drugs, including S-1, capecitabine and FU itself. S-1 is a fourth generation oral fluropyrimidine prodrug that 5-chloro-2, 4-dihydropyrimidine (CDHP, a dihydropyrimidine dehydrogenase inhibitor) and potassium oxonate, part of which translates into FU after entering human body [30], while capecitabine is an oral fluropyrimidine, which can also be metabolically converted into FU in the body. Although no significant statistical difference was detected among the three drugs in treating gastric cancer [31, 32], it remains unclear whether they have different effects on patients with BTC. Second, the treatment TAR also included several targeted drugs, including cetuximab, erlotinib, panitumumab, vandetanib, cediranib and sorafenib. The effects of targeted drugs may differ from each other. For example, a study conducted by Moehler [19] reported that the addition of sorafenib to GEM did not demonstrate improved efficacy while as mentioned before, the survival effect of cetuximab has been confirmed in previous study [9]. To further analyze the effect of different targeted drugs, more detailed classification should be made in future studies. Thirdly, PLA in this NMA includes CIS and OXA. In fact, the difference has already been reported between GEM+ CIS and GEM+OXA [33], which may result in the heterogeneity of this study. Finally, the lack of evidence led to some missing information of some treatments. For example, 3 out of 4 survival outcomes were missing with respect to the treatment of GEM+TAR. Therefore, further effort should be made in order to gain a more comprehensive result.

In conclusion, the addition of PLA can significantly improve the efficacy of FU and GEM-based treatments, and the addition of TAR to GEM+PLA can contribute to further improvement, but with a mild increase of adverse events. Thus, GEM+PLA and GEM+PLA+TAR are both recommended and the option is depended on the conditions of patients. As the first article for the comprehensive comparison of different chemotherapy treatments for BTC, our study could be served as a reference for clinical treat. Furthermore, more detailed analysis should be conducted in order to gain a more comprehensive result.

## MATERIALS AND METHODS

### Literature search

PubMed, Embase, Cochrane Library were searched for potentially eligible publications of related diseases. The following key terms and their synonymous terms were used, including “biliary tract cancer”, “fluorouracil”, “gemcitabine”, and “targeted medicine”. In addition, reviews whose data was available were also included. The searching procedure was accomplished by two investigators independently.

### Selection criteria

Eligible studies should meet the following criteria for further analysis: 1) patients should be diagnosed with advanced or unresectable BTC; 2) studies should include at least two of the followingtreatments: BSC, FU, PLA, GEM, TAR; 3) at least one of the included efficacy and adverse effect outcomes should be reported. 4) Studies were RCTs investigating the.comprehensive efficacy and adverse effects of treatments for BTC. Besides, we excluded the duplicate experiments, reviews and case reports from previous analysis and articles.

### Data extraction

Two investigators conducted data extraction respectively. By scanning the eligible records, the fundamental information of these trials was abstracted, including the last name of the first author, publication year, country, study design, follow-up period, treatments, age of patients, male proportions, sample size and outcomes.

### Statistical analysis

Network plot was computed in order to illustrate the comparisons from the included records. The size of each circle represents the sum of the samples; a solid line represents the direct comparison of the two therapies and the width of each line stands for the number of two-arm trials comparison.

Next, a Bayesian framework and *Markov Chain Monte Carlo* (MCMC) simulations NMA was applied. R 3.2.3 software and STATA 13.0 were used to conduct this analysis. Based on random effects model, hazard ratios (HRs) and 95% credible intervals (CrIs) were calculated to compare the effects on long-term survival of each different treatments for OS and PFS. While for ORR and DCR, odd ratios (ORs) combined with 95% CrIs were used to assess the pharmacological effects. Moreover, four adverse events, including vomiting, nausea, neutropenia and thrombocytopenia, were analyzed in terms of OR with corresponding 95% CrIs to evaluate the relative safety of included treatments. In the end, surface under the cumulative ranking curve (SUCRA) was calculated to obtain the rankings of different BTC treatments.

## Abbreviations

BTC: biliary tract cancer
GBC: gallbladder cancer
ICC: intrahepatic cholangiocarcinoma
ECC: extrahepatic cholangiocarcinoma
HCC: hilar cholangiocarcinoma
BSC: best supportive care
GEM: gemcitabine
FU: fluorouracil
TAR: targeted drugs
OXA: oxaliplatin
CIS: cisplatin
PLA: platinum
OS: overall survival
PFS: progression free survival
ORR: overall response rate
DCR: disease control rate
